# Assessing the Impact of Customary Ownership of Trees and Socioeconomic Factors on the Participatory Forest Management in Jharkhand, India

**DOI:** 10.1007/s00267-025-02129-x

**Published:** 2025-02-22

**Authors:** Sabyasachi Kar, Mukti Ram Subedi, Parag Kadam, Puneet Dwivedi

**Affiliations:** 1 Department of Forestry and Environmental Conservation, Clemson University, Clemson, SC 29634 USA; 2 Warnell School of Forestry and Natural Resources, University of Georgia, Athens, GA 30602 USA

**Keywords:** Community forest management, Participation, Tree rights, Customary rights, Sustainable development

## Abstract

Customary ownership over trees on forested public lands is a traditional practice that grants individuals or groups rights and duties to access, use, and manage trees. This unique ownership model, where the Indian Government owns the forested land, but trees on that land are customarily owned by the households or community, plays a critical role in community participation in Participatory Forest Management (PFM). No study has yet quantitatively analyzed the relationship between customary ownership over trees and participation in PFM activities. Therefore, this study fills this gap by analyzing the relationship between households’ participation in PFM, the number of trees under customary ownership on forested public lands, and socioeconomic variables in Jharkhand, India. Data were collected through semi-structured interviews and participatory rural appraisal. Factor analysis and multivariate linear regression were employed to analyze this relationship. We found that households’ customary ownership over a higher number of trees enabled them to earn a higher income and motivated them to relocate for better education and healthcare access, resulting in reduced PFM participation. Households with larger forest landholdings participated more regularly in PFM activities, motivated to legalize ownership of their long-used forestlands. Displaced households experienced reduced PFM participation. Higher caste and wealth groups were associated with greater PFM participation due to better resource access and stronger decision-making power. The study findings inform policymakers to improve inclusive participation in PFM activities and provide a pathway for achieving India’s environmental commitments by reducing socioeconomic disparities, improving tribal livelihoods, and promoting sustainable management of forestry resources.

## Introduction

The centralized forest governance regime of the colonial period and the early years of India’s independence have disrupted the symbiotic relationship of tribal and local people with their forests (Mahapatra 2002; Tripathi 2016). Additionally, the governments have introduced policies and implemented development projects that have often displaced forest-dependent communities (Banerjee and Madhurima 2013; Baviskar 1994; Gadgil 1990). Specifically, between 1947 and 1997, development projects such as dam construction, roads, mining, and railway expansion displaced more than 50 million people (Fernandes 1998; Mahapatra 1999; Ramakrishnan 2007). Approximately half of those displaced communities were Scheduled Tribes, who mainly reside in hilly and forested regions where most development projects are located (Baviskar 2019; Venkteshwar 2023). Furthermore, these displacements have done damage to the deep-rooted connections of forest-dwelling communities with the forest, resulting in economic hardship and social marginalization (Negi and Ganguly 2011). These disturbances led to widespread community movements emerging across regions, from the Himalayan ranges to the tropical forests of Kerala and from Gujarat in the west to Tripura in the northeast (Karan 1994). These movements ranged from the Chipko Movement in Uttarakhand, the Narmada Bachao Andolan in Madhya Pradesh and Gujarat to the Appiko Movement in Karnataka, the Niyam Giri Hills movement of Odisha, and the Gumti Dam movement in Tripura against ecological degradation and social dislocation (Karan, 1994).

In response to these grassroots movements, the Government of India implemented pilot projects in West Bengal, Madhya Pradesh, and Haryana, which paved the way for participatory forest management (PFM) programs and policies in India (Bhattacharya et al. 2010). The Government of India formally introduced PFM through the 1988 National Forest Policy (NFP). PFM was introduced as an approach that differs from centralized and conservation models that assume a separation between humans and nature (Bawa et al. 2021; Madhusudan and Raman 2003). It actively involves local forest-dependent communities in forest management and conservation-related decision-making processes (Ribot et al. 2010; Okumu and Muchapondwa 2020). The Food and Agricultural Organization (FAO) defines PFM as ‘the process that involves people who have a direct interest in forest resources in all aspects of forest management.’ This includes decision-making, policy formulation, and management of resources (Moss et al. 2005).

PFM in India has undergone many changes over several decades through the introduction of new programs and policies (e.g., the 1990 Joint Forest Management (JFM) program and the 1995 Panchayat Extension to Scheduled Areas (PESA) Act). These policies aim to balance the well-being of humans and forests by acknowledging the interests of marginalized communities and valuing their traditional knowledge, customary rules, and caretaking practices related to forest management (Dhanapal 2019; Nagahama et al. 2022). They also acknowledge the vital role of forest-dependent communities in forest conservation and protection (Springate-Baginski and Blaikie 2007). The 2006 Forest Rights Act (FRA) introduced a significant shift toward participatory approaches against the backdrop of historical injustices to forest-dwelling communities belonging to Scheduled Tribe categories and other traditional forest-dwelling peoples. It emphasized their crucial role in the sustainability of the forest ecosystem.

PFM offers both tangible benefits, such as timber, energy, fodder, and non-timber forest produce, and intangible benefits, including soil fertility, nutrient cycling, and watershed management, while supporting livelihoods through the sale of forest produce (Raj et al. 2021). Studies show that PFM improves forest conditions, driving rural development, alleviating poverty, and ensuring gender equity (Blomley et al. 2008; Borgoyary et al. 2005; Jana et al. 2014). It also empowers communities to sustainably manage their forests by tackling the challenges of illegal logging, forest fires, open access, social and environmental justice, and promoting conservation (Blaikie and Springate-Baginski 2013; Winberg 2011).

Different factors determine the participation of community members in PFM. To understand the determinants of participation in PFM comprehensively, we reviewed several studies over time and geographic scales, including studies focused on India (Table 1) and studies focused on regions outside India (Table 2). We found that the factors related to the success of PFM are favorable policy regimes, conducive property rights, and the intricate system of land tenure arrangements (Basu 2021; Baynes et al. 2015; Berkes et al. 1989; Ghosh and Basu 2021). Furthermore, gender, caste, class, household size, level of household head, economic status of the household, land holdings, distance from forest, number of livestock holding, availability of common and forestlands, and forest dependency also affected participation in various PFM-related activities (Bandyopadhyay and Shyamsundar 2004; Bista et al. 2023; Heltberg 2001; Jana et al. 2014; Jatana and Paulos 2017; Nandigama 2020).

**Table 1:** Analysis of the determinants of participation in forest management activities in India using various independent variables and methodologies

Study area	Variables influencing PFM participation	Model	Source
West Bengal, India	Caste and sex of the respondent, Age of the head of households, Occupation of the head of households, Landholdings of the households, Monitoring Index	MLR	Ghosh and Basu (2021)
West Bengal, India	Caste of the household, Family size, Gender of household head, and Educational Index are measured based on UNDP methodology, Number of occupations, land holdings, % of forest income to total income (monthly), Distance between residence and forest, Distance between residence and market, Cooperation from forest authority.	MLR	Basu (2021)
West Bengal, India	Respondent’s years of education and Gender; Household size, religion, number of meetings, Willingness to pay for the forest, Land ownership, Forest dependence, Consumption per capita (household consumption divided by household size), Capital per capita (household capital divided by household size).	PCA, MLR, and Game theory	Jana et al. (2014)
Andhra Pradesh, India	The total population in the community forest has valuable tree species, Number of active community-based organizations in the village, Population per hectare of JFM forest areas, and Access rights over JFM forest resources.	OL	Behera (2009)
Jharkhand, Orissa and West Bengal, India	Leadership styles and attributes of leaders (manipulative, authoritarian, participatory, charismatic, members’ closeness to the leader, leaders’ virtues, Maintenance of relationships, Idealized behavior, direct participation, indirect participation).	Stepwise regression, Post hoc analysis using Tukey’s Test	Sinha and Suar (2005)
Andhra Pradesh, Madhya Pradesh, Orissa, Uttar Pradesh, and West Bengal, India	Amount of fuelwood used for consumption & enterprise, Caste, Level of reading newspaper, Total Village Common Land (ha), Proportion of participating households in Community Forestry, Fuelwood Price (Rs/Kg), Non-Agricultural Labor.	Binomial probit model, and MLR	Bandyopadhyay and Shyamsundar (2004)
Rajasthan, India	The size of the user group is measured as the log of the village population, resource scarcity is measured as the log of the village population relative to the area of forest and commons, and infrastructure development is measured with the Development Index and the existence of a Temple Land.	Logistic regression	Heltberg (2001)
Haryana, Uttar Pradesh and Bihar, India	Caste and religion of the respondent, Forest dependence, Years of schooling of the respondent, Consumption per capita (rupees/year), Income per capita (rupees/year), and Capital per capita (rupees).	Factor analysis and MLR	Lise (2000)

*MLR* multiple linear regression, *OL* ordered logit regression, *OPM* ordered probit model, *PCA* principal component analysis

**Table 2:** Analysis of the determinants of participation in forest management activities outside India using various independent variables and methodologies

Study Area	Variables influencing PFM participation	Model	Source
Middle Hills of Nepal	Total size of household, Total number of internal migrants, Caste of HH head, Walking distance to the forest in minutes, Training opportunity, Executive Committee position	OL	Bista et al. (2023)
Mandalay region, Myanmar	Nonfarm income (remittance, casual labor (non-farm), small business, nonfarm salary jobs), Family working outside of township area (Households have at least one household member working outside of township area at least 6 months in a year), Family labor (Working age household member (16–62 years old))	Binary logistic regression	Maung, Masahiro (2023)
Oromia regional state, Ethiopia	Forest dependence, Farm size (in ha), Number of male adults, Number of local organizations	Mixed effect linear regression	Gatiso (2019)
Wolaita zone, Southern Ethiopia	Annual income, Household head’s age, Land size, Sex of the respondent, and household size	Logistic regression	Jatana and Paulos (2017)
Western Nepal	Gender and Caste of the respondent, Household Size, number of livestock owned by the respondent’s household, Total number of social groups to which household is attached, firewood received from the community forest over the past year	OPM	Oli and Treue (2015)
Burkina Faso, West Africa	Gender and Marital status of the respondents, Household size, technical assistance received from organizations, Participation in voluntary organizations, Forest policy, and forest legislation	Factor Analysis and MLR	Coulibaly-Lingani et al. (2011)
Bartın province, Turkey	Age, marital status, and number of pregnancies of the respondent, Source of family income, Number of alternatives for providing home water, Number of tools for cooking, Benefits from forest resources, Monthly income of family, Forest area per capita	PCA and MLR	Atmiş et al. (2007)
Borno, Kano, and Sokoto, Northern Nigeria	Age, Monthly income, Farm size, Tangible infrastructure asset	MLR	Oloruntoba and Adeola (2006)
Haiti	Respondent’s age, Respondent’s gender, Respondent’s labor status, Respondent’s benefit from the forest, Respondent’s years of education, Respondent’s member of local group, Respondent’s tenure status, Technical Assistance received from Ministry of Agriculture.	Factor analysis and MLR	Dolisca et al. (2006)

*MLR* multiple linear regression, *OL* ordered logit regression, *OPM* ordered probit model, *PCA* principal component analysis

Despite its potential, challenges still exist even after four decades of implementing PFM across India, despite many well-intended policies. These challenges include institutional gaps, socioeconomic inequalities, gender and caste disparities, bureaucratic resistance, limited decision-making power to local communities, overemphasis on monoculture plantations affecting biodiversity, and the inability to integrate social, ecological, and economic dimensions into sustainable forest management frameworks (Dhanapal 2019; Nandigama 2020; Nagahama et al. 2022). Another critical challenge in the PFM is the ambiguity in the legal rights and tenure system. The 1927 Indian Forest Act (IFA) provides property rights over forest land to the Government of India (IFA 1927); however, the question “Who owns trees on forested public land?” remains unanswered. This question becomes important as many studies highlight that land ownership and tree ownership are distinct. For example, Kar et al. (2024) and Kabra et al. (2023) highlighted that Santhal households in Jharkhand and Sahariya households in Madhya Pradesh claim customary ownership rights over 18,000 and 85,000 trees, respectively, on state-owned forestlands. This kind of ownership model includes the right to own or inherit trees, the right to plant trees, the right to use trees and tree products, the right to dispose of trees, and the right to exclude others from the use of trees and tree products and are governed by customary governance systems and shaped by ecological, social and legal factors (Fortman 1985). Customary ownership over trees on forested public land are deeply rooted within the community and go beyond mere possession of trees (Howard and Nabanoga 2007; Kabra et al. 2023). Customary ownership over trees, whether communal, shared, or individual, is influenced and shaped by factors such as religion, economic status, access to forestlands, kinship, gender, and land tenure systems (Fortman 1985; Kar et al. 2024). It can promote the well-being of forest-dependent communities by preventing exploitation while ensuring sustainable management of forest resources (Fortman 1985; Garcia et al. 2010). In addition, customary rules shaping this kind of tree ownership greatly influence community participation in forest management activities (Barrow et al. 2016; Takahashi et al. 2024).

Although the literature highlights that customary ownership over trees on forested public land is prominent in many tribal societies and fosters social and spiritual bonds among forest-dwelling tribal communities, there has been no quantitative exploration of the relationship between ownership of trees on forested public land and household participation in PFM, with the assumption that tree ownership is tied with land ownership (Howard and Nabanoga 2007). Additionally, the 2006 FRA recognizes individual forest rights, community forest rights, and community forest resource rights and grants forest land rights along with other customary rights but does not explicitly acknowledge customary ownership over trees on forested public lands (FRA 2006; Ballal et al. 2023). In this context, our study seeks to answer the research question: How does the number and location of trees on forested public land under customary ownership influence households’ participation in PFM? What are the impacts of socioeconomic variables on household participation in PFM? How does the interaction between customary ownership tree ownership and socioeconomic factors contribute to the participation of households in PFM? To address these questions, this study aims to analyze the influence of the number and location of trees on forested public land under customary ownership on household participation in PFM activities, considering socioeconomic covariables, such as caste, income, landholding, and displacement.

Our study is highly relevant in the context of current policy. On the one hand, the Government of India has introduced the 2023 National Working Plan Code (NWPC) and the 2014 National Agroforestry Policy (NAP) to achieve the goal of the 1988 National Forest Policy of 33% forest cover of India’s total geographical area (NFP 1988). These initiatives aim to fulfill India’s international commitments through PFM. They emphasize the recognition of traditional knowledge and customary systems and the integration of trees with agricultural practices to improve productivity and support the livelihoods of forest dwellers (NWPC 2023; NAP 2014). On the other hand, the Government of India has introduced the 2023 Forest (Conservation) Amendment Act (FCA), which narrows down forest definitions and undermines communities’ governance and management rights over community forest resources (Ramakrishnan 2024; Saxena 2024; Ballal et al. 2023). Recognizing the importance of customary ownership over trees on forested public lands can bridge this gap, serving as a critical link to strengthen PFM strategies and ensure alignment with NWPC and NAP goals for sustainable forest management, community empowerment, and achieving international commitments.

## Methods and Materials

### Study Site

**Fig. 1:**
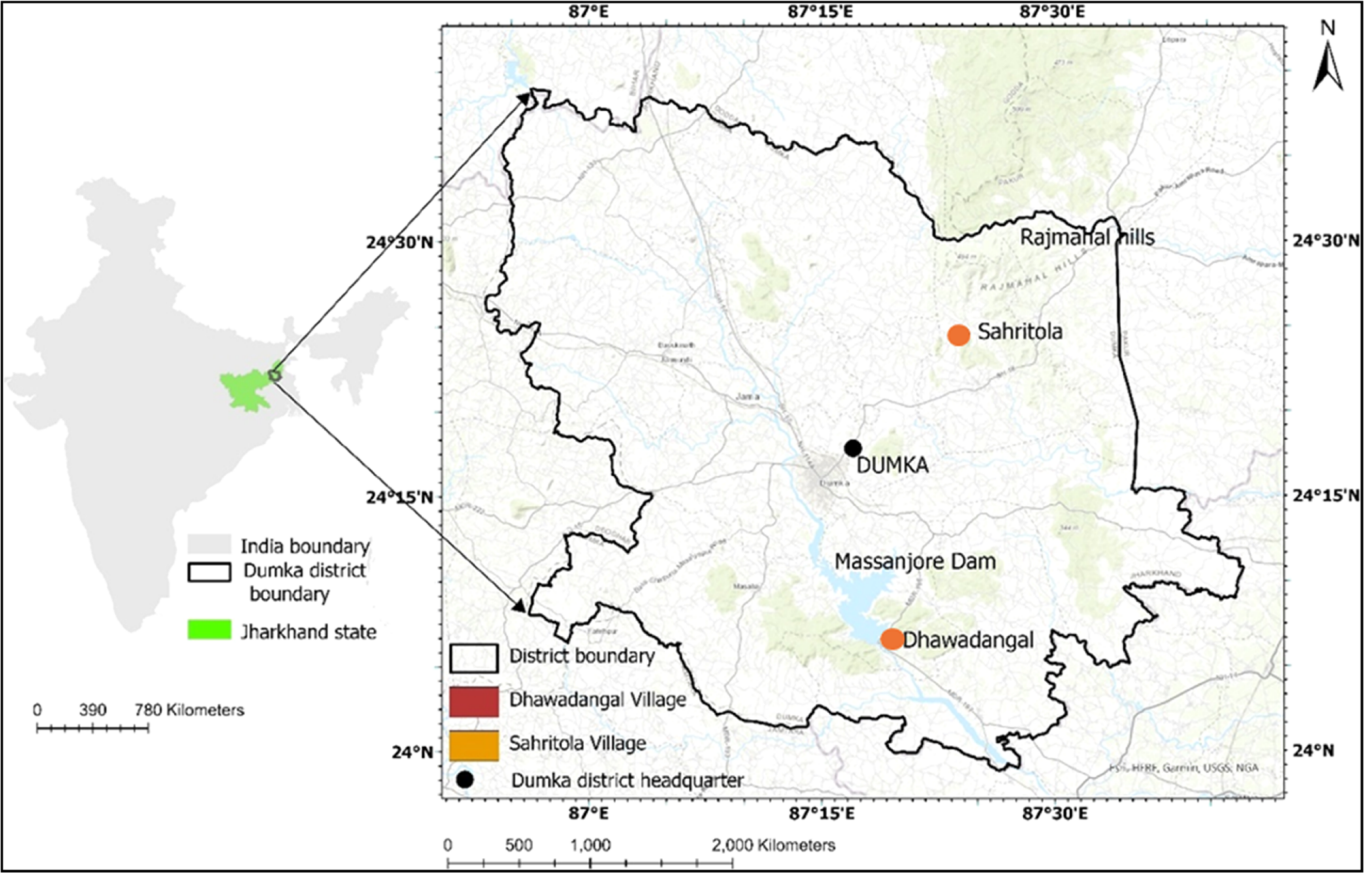
Location map of the studied villages, Sharitola and Dhawadangal, in Jharkhand, India. Basemap Sources: ESRI, DeLorme, HERE, TomTom, Intermap, increment P Corp., GEBCO, USGS, FAO, NPS, NRCAN, GeoBase, IGN, Kadaster NL, Ordnance Survey, Esri Japan, METI, Esri China (Hong Kong), swisstopo, MapmyIndia, and the GIS User Community

The study was conducted in Dhawadangal and Sahritola, in Dumka District, Jharkhand, India (Fig. 1). These villages are situated within the Rajmahal hill range near the Massanjore Dam. The construction of the Massanjore dam resulted in large-scale displacements of the Pahariya communities (Particularly Vulnerable Tribal Groups, PVTG), who were resettled in nearby villages, including Dhawadangal (Rao 2005). A Particularly Vulnerable Tribal Group or PVTG is a sub-classification of Scheduled Tribe. PVTGs are more vulnerable than a regular Scheduled Tribe with a declining or stagnant population, low level of literacy, practices pre-agricultural level of technology, and are economically backward. Seventy-five such groups of Tribals mostly reside in 18 states and 1 Union Territory (Ministry of Tribal Affairs, Government of India (2015)).

The area is characterized by dry deciduous forests, with the main tree species available in the forests being sal (*Shorea robusta*), asan (*Terminalia tomentosa*), mahua (*Madhuca longifolia*), simul (*Bombax ceiba*), and teak (*Tectona grandis*). The population in both villages mainly comprises the Santhal and Mal Pahariya communities and other backward classes. Their livelihoods are primarily based on traditional subsistence agriculture, seasonal migration to urban employment, and forest-related activities such as sericulture, collecting medicinal plants, and collecting and selling forest products such as fruits and flowers. The Santhal, an indigenous community in India, is organized into 12 totemistic clans and 164 subclans, practices patrilineal descent, maintains an egalitarian society, relies on oral traditions, and possesses a rich body of indigenous knowledge that supports their cultural identity (Carrin-Bouez 1998). The Mal Pahariya community, categorized as a PVTG, originally inhabitants of the Rajmahal Hills, known today as the Santhal Parganas division of Jharkhand as well as some districts of West Bengal. They belong to the Dravidian stock. Additionally, some scholars classify them as Proto-Australoid, indicating their ancient anthropological lineage (Bandyopadhyay 2022).

### Data Collection

The study was conducted between January 2023 and August 2024, and the fieldwork for data collection was performed between June and August 2023. There was a total of 156 households in the Sahritola and Dhawadangal villages. Before data collection, we conducted two public meetings in two villages to explain the study objectives. Each meeting was attended by 20–30 participants, including village residents (both men and women) and village heads. During these meetings, we finalized a list of 144 participating households out of 156 households in two villages. In this study, we tried to include all 156 existing households in two villages; however, 12 households were absent during data collection, as they went to other nearby cities for work. The sampling approach was exhaustive, targeting the entire population of the two villages, and the participation was based on the availability and willingness of the household heads. Before data collection, we obtained consent from each participating household according to the institutional review board protocols of the University of Georgia. We also clarified that no compensation would be provided for survey participation. Data collection involved Participatory Rural Appraisal (PRA), household interviews, and geospatial mapping of households and their trees in nearby forested public lands.

In each village, we facilitated discussions with 25–30 individuals, including men and women, to conduct a PRA. Initially, these participants classified other participating households into four wealth groups (rich, better off, poor, and very poor) based on their socioeconomic status. The socioeconomic status of the households was determined based on the criteria outlined during the PRA exercise, as detailed in the supplementary material (Table S1). These criteria included indicators such as food security status, land ownership, livestock holding, income sources, and annual income. These indicators were used to classify households into wealth groups. Subsequently, we combined rich and better-off groups into a single category labeled better-off. Of the 144 households that participated in this study, 48 households were classified as better-off, 32 as poor, and 64 as very poor.

**Fig. 2:**
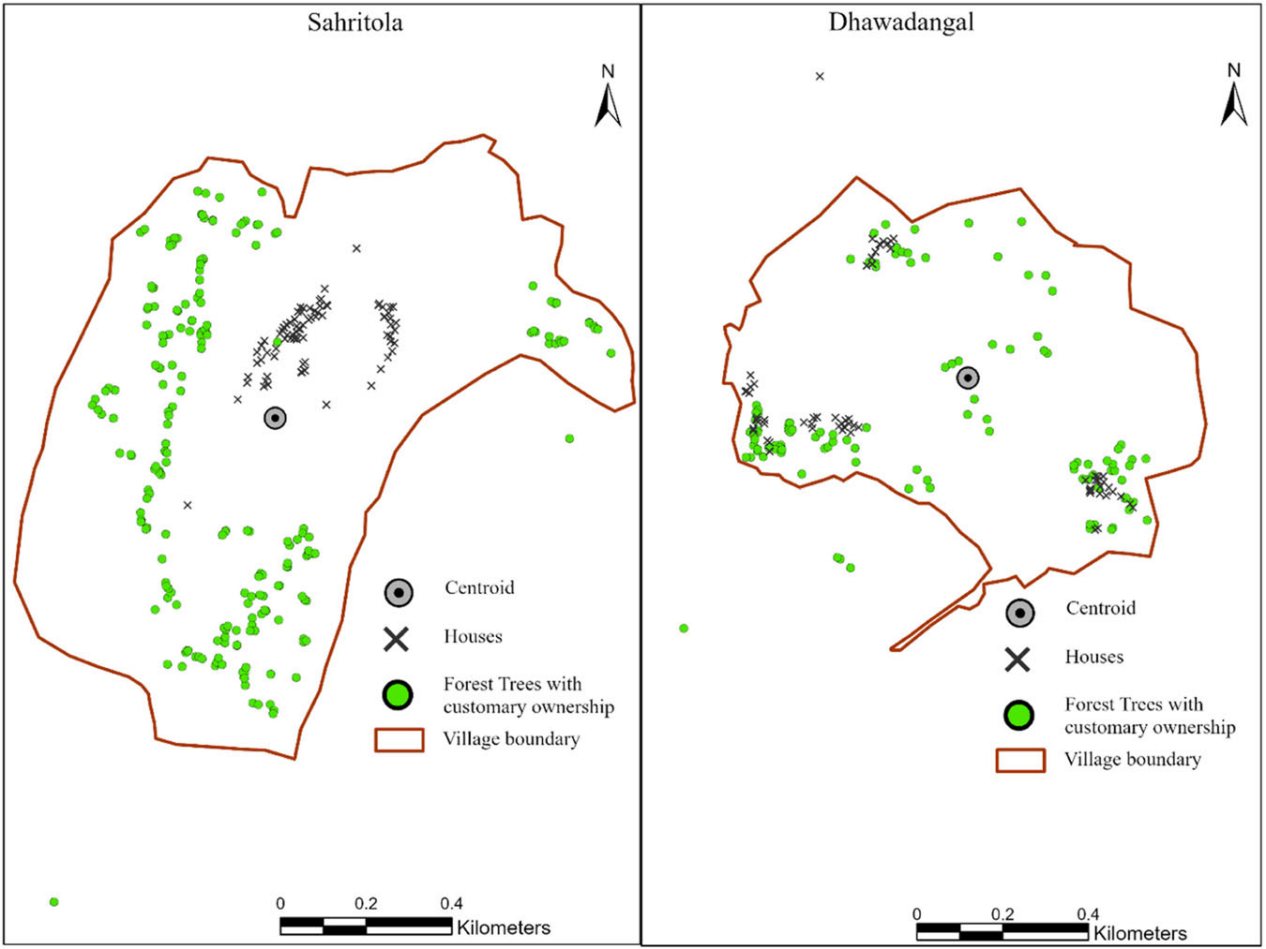
A map of the selected villages showing the locations of the houses. A circle with a black dot in the center indicates the geometric centroid of the village. The green points indicate the location of trees with customary ownership

We designed a semi-structured household questionnaire to collect data on the socioeconomic dimensions and details of customary ownership of the households. The questionnaire covered demographic profile, land holding size, primary and secondary income sources, the total number of trees owned on forested public lands under customary ownership, and participation in various PFM activities, such as attending forest committee meetings, participating in decision-making processes, and contributing to forest-related tasks. We used PRA Wealth Ranking tools in two villages to assess the economic status of the households and categorized them (Table S1) into three categories: better-off, poor, and very poor (Adams et al. 1997; Chambers 1994). The geospatial coordinates of the houses of all households were collected using Nautiz X6 and Environmental Systems Research Institute Inc. (ESRI) Field Map (Fig. 2) (ArcGIS 2023). Additionally, we recorded the locations of trees owned by each participating household on forested public lands, if applicable.

### Measurement of Household Participation in Forest Management

In the present study, we define participation (a dependent variable) following Agarwal (2001) and Tyagi (2021) as participation in an activity when asked or voluntarily (activity-specific participation), expressing opinions or taking the initiative (active participation), and having a voice in the group decision-making process (interactive/empowering participation) (Table 3). To measure participation, respondents were asked the following questions: (a) How often do households attend forest committee meetings? Please note that forest committees were formed by a local non-profit to implement the provisions of the 2006 FRA. (b) To what extent do households participate in emergency events to mitigate the situation? Here, emergency events refer to situations that directly affect the condition of the forest. These include incidents such as forest fires and illegal logging by outsiders. (c) To what extent do households participate in forest protection activities and follow recommended practices? (d) During conflict resolution on forest resources, how often do households share their opinions? These responses were recorded on three Likert scales, i.e., frequently, moderately, and rarely. To ensure a uniform understanding of these terms, we defined them and provided a context-specific example to each respondent. ‘Frequently’ was defined as attending almost all forest committee meetings organized by the non-profit and the forest department, participating in almost every forest-related emergency situation, sharing opinions in every relevant situation, and helping to resolve conflicts. It also refers to participation in forest protection activities, such as social guarding and patrolling during the household’s turn. ‘Moderately’ was defined as attending some forest committee meetings, participating in emergency events occasionally, sharing opinions in selected situations, and contributing less frequently to forest protection activities. ‘Rarely’ was used to indicate that households did not participate in these activities or did so on rare occasions.

**Table 3:** Description, abbreviation, and type of variable used in factor analysis and multiple linear regression

Description	Abbreviation	Type
1. How often do households attend forest committee meetings? Scale [1–3]	attend_meeting	Ordinal
2. To what extent do households participate in emergency events to mitigate the situation? Scale [1–3]	emgr_evnt	Ordinal
3. During conflict resolution concerning forest resources, how often do households share their opinions? Scale [1–3]	opinion_sharing	Ordinal
4. To what extent do households participate in forest protection activities and follow recommended practices? Scale [1–3]	forst_activities	Ordinal
5. Number of trees with customary rights located on government-owned forestland.	forstree	Discrete
6. Total household members	tothh	Discrete numerical
7. Age of the head of household in years.	agehhhd	Discrete numerical
8. Number of school years attended in years by the household head.	eduhhhd	Discrete numerical
9. The household’s average age (years) was calculated as the sum of all members’s ages divided by the total number of household members.	avghhage	Continuous
10. Total agricultural land in ha with legal title.	agrland	Continuous
11. Total forest area (ha) used by households from generation to generation for either shifting cultivation, silkworm farming, or NTFP collection.	forsland	Continuous
12. Total title land(ha), including agricultural land, barren land, homestead land, or any other land mentioned in their land title document	titledland	Continuous
13. Euclidean distance (m) between houses and forest trees under customary ownership	treedist	Continuous
14. Hindu, coded as 1, and Christian - coded as 2	religion	Categorical
15. Scheduled Tribe - coded as 1, Particularly Vulnerable Tribal Groups (PVTG) - coded as 2, Other Backward Classes - coded as 3	caste	Categorical
16. The economic status was determined through a wealth ranking activity, a PRA tool. This activity identifies three categories: Better-off (3), Poor (2), and Very Poor (1).	econ	Categorical
17. Identified four main categories of income sources that contribute primarily to household income: agriculture (1), forest-based activities (2), wage labor and migration (WM) (3), and other (e.g., services, income from social welfare schemes, and livestock rearing) (4).	livelihood	Categorical
18. Households displaced from their original village during their lifetime are coded as 1 (Yes), and those that have not are coded as 0 (No).	displace	Categorical

(*N* = 144)

In addition, the demographic and socioeconomic characteristics of the respondents were collected. These included the number of trees under customary ownership (forstree), household size (tothh), the average age of the household (avghhage), age of the household head (agehhhd), education status (number of school years attended in years) of the household head (eduhhhd), agricultural land holding (agrland), forestlands using (forsland), total land with the title (titledland), the distance of those trees from their owners’ residences (treedist), religion (religion), caste (caste), economic status (econ), primary livelihood (livelihood) and displacement status (displace) (Table 3).

### Data Analysis

Data collected from a semi-structured questionnaire survey on household participation and socioeconomic data were analyzed through descriptive and inferential techniques. Specifically, we used exploratory factor analysis (EFA) and ordinary least squares (OLS) regression. We first performed an EFA, and the results obtained were then used in the OLS regression associating socioeconomic variables. The Euclidean distance of forest trees under customary ownership from their owners’ residences was calculated using ArcGIS Pro (v 3.3) (Esri. 2022). All other analyses were performed in R statistical software R (R Core Team 2013) using packages ‘psych’ (Revelle 2024), ‘lavan’ (Rosseel, 2012), and ‘stats’ (R Core Team 2013). Nonparametric Kruskal-Wallis tests were conducted to examine (1) differences in the number of trees under customary ownership across various wealth groups and (2) participation in PFM activities across varying economic statuses, followed by pairwise comparison using the Wilcoxon rank test with continuity correction to identify specific group differences.

### Factor Analysis

We performed factor analysis to reduce the number of variables affecting household participation in PFM. New factorial dimensions were produced to minimize variance loss according to the correlation matrix on the original participation variables. The factor analysis allowed us to summarize the information, highlighting the latent relations among the original participation variables. The resultant factors produced can be used in other analyses, such as OLS (Dolisca et al. 2006; Lise 2000). To assess the goodness of fit of the EFA, we performed the Kaiser-Mayer-Olkin (KMO), Barlett’s test, and Tucker Lewis Index (TLI). The KMO test compares the observed and partial correlations between the variables in the model and produces index values between 0 and 1. Generally, a KMO test value higher than 0.7 indicates good reliability. We also used Barlett’s test to test the null hypothesis that the correlation matrix coincides with the identity matrix, assessing whether the total correlation of the model is 0. A significant Barlett’s test indicated a meaningful relationship among the matrices, showing that the observed correlations are not due to random chance and that factor analysis is appropriate. The total number of factors to be retained was determined through the scree plot. The scree plot suggested only one factor; therefore, factor extraction was performed using the maximum likelihood estimation without rotation.

### OLS Regression

To assess the significance of the extracted participation factor through EFA, an OLS regression was created to establish the relationship between participation and socioeconomic variables. We scaled and applied cube root transformation on the extracted factor for this. Several predictors were used, including the number of trees under customary ownership, household demographics, land holdings, and various categorical variables such as religion, caste, economic status, livelihood, and displacement. The resultant model is represented by below equation.

1 $$\begin{array}{ll}Participation \, = \, {\beta }_{0}+{\beta }_{1}\cdot {\log }_{10}(forstree)+{\beta }_{2}\cdot tothh+{\beta }_{3}\cdot avghhage\\\qquad\qquad\qquad\qquad+ \,\, {\beta }_{3}\cdot agehhhd+{\beta }_{4}\cdot eduhhhd+{\beta }_{5}\cdot agrland+{\beta }_{6}\cdot forsland\\\qquad\qquad\qquad\qquad+ \,\, {\beta }_{7}\cdot titledland+{\beta }_{8}\cdot treedist+{\beta }_{9}\cdot religion+{\beta }_{10}\cdot caste\\\qquad\qquad\qquad\qquad+ \,\, {\beta }_{11}\cdot econ+{\beta }_{12}\cdot livelihood+{\beta }_{13}\cdot displace+{\varepsilon }_{i}\end{array}$$

where Participation is a cube-root transformed factor obtained from EFA analysis. ${\beta }_{0}$ is intercept term, ${\beta }_{1}-{\beta }_{13}$ are slopes for independent variables. The independent variables are defined in Table 3. ${\varepsilon }_{i}$ is the error terms with iid ~N(mean, variance).

## Results

### Profile of the respondents and village forest

Within the villages studied, the average size of the household (tothh) was four, with an average age of the household (average age) of 29 years (Table 4). The average age of the heads (avghhhd) was 42 years, and their levels of education (eduhhhd) were relatively low, averaging four years of formal education. The respondents owned an average of 0.30 ha of agricultural land (agrland), 0.20 ha of forest land used (forsland), and 1 ha of titled land (titledland). Please note that Agricultural land (agrland) means total agricultural land in ha with legal title. Total Titled land (titleland) in ha, including agricultural land and nonagricultural lands such as barren land, homestead land, or any other land mentioned in their land title document. The average Euclidian distance between customarily owned forest trees and their owners’ residences was around 400 m. The community predominantly followed the Hindu religion (72%), and 28% followed the Christianity. About 57% of the households were Scheduled Tribes, particularly Santhals, 35% were Mal Pahariyas, and the rest belonged to other backward classes. Forty-five percent of the respondents relied primarily on forest-based activities such as cultivating tasar silkworms and collecting mahua flowers as income. At the same time, 28% depended on migration to urban cities to work. Socioeconomically, a significant portion of households were classified as very poor (44%), followed by poor (22%) and better off (33%). Approximately one-third of the respondents had been displaced due to the construction of the Massanjore dam (32%).

**Table 4:** Descriptive statistics of continuous variables (*N* = 144)

Variable	Minimum	Maximum	Mean	Standard deviation (SD)
Number of forest trees under customary ownership	0	3064	128	292
Total household members	1	10	4	2
The average age of the household	12	68	29	13
Age of household head	20	80	43	14
Number of school years of household head	0	15	4	4
Agricultural land holding	0	1.7	0.3	0.3
Forestland used	0	1.3	0.2	0.2
Total titled land holding	0	8.1	1.0	1.1
Distance of forest trees under customary ownership from the residences	0	1633	515	360

**Fig. 3:**
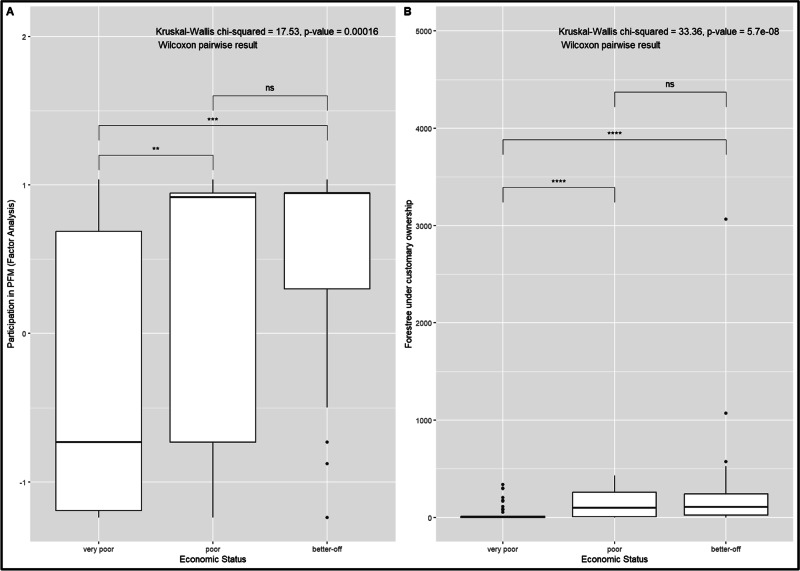
Participation in PFM activities and the number of forest trees under customary ownership among various economic status groups were analyzed using the Kruskal-Wallis nonparametric test and Wilcoxon pairwise comparisons with continuity correction. **A** For participation in PFM activities derived from factor analysis by economic status, significant differences were found between very poor and better off (*p* < 0.001) and very poor and poor (*p* < 0.01). **B** Distribution of forest tree ownership among varying economic status, showing highly significant differences between very poor and poor and better off (*p* < 0.0001)

Most households (54%) reported participating in forest committee meetings ‘moderately,’ with 28% of households stating that they attend meetings frequently, while 18% of households noted that they rarely attend meetings (Table 5). In emergencies such as forest fires and illegal logging, 44% of households frequently managed the situation, but 17% rarely participated. Regarding forest protection activities, most (52%) adhered to recommended practices, while 20% rarely participated. During the resolution of forest resource conflict, a significant proportion (51%) of households frequently shared their opinion, compared to 21% who rarely did. These findings highlight the different degrees of participation in PFM activities. Furthermore, the Kruskal-Wallis test result indicates a significant difference in participation in PFM activities by economic status (*p* < 0.001) (Fig. 3A). Pairwise comparisons using the Wilcoxon rank sum test show significant differences in participation between households with very poor and households with better income (*p* < 0.001) and very poor households and poor (*p* < 0.01) households.

**Table 5:** Household participation levels in forest management activities *(N* = 144)

Management activity	Rarely	Moderately	Frequently	Factor loadings
Attending meeting (attnd_meet)	18%	54%	28%	0.77
Participate in emergency events (emgr_evnt)	17%	38%	45%	0.95
Opinion sharing during conflict resolution (opinion_sharing)	21%	28%	51%	0.99
Participation in forest protection activities and follow recommended practices (forst_activities)	20%	28%	52%	0.92
Percentage of variance				83%
eigenvalue for the single factor				3.32

The responses were recorded on a Likert scale: ‘frequently’ (active and regular participation in meetings, emergencies, and forest protection), ‘moderately’ (occasional participation in these activities), and ‘Rarely’ (minimal or no participation)

We observed a significant disparity in trees under customary ownership on forested public lands between various economic statuses (Table 6). Better-off households customarily owned 60% of the trees under customary ownership. The average number of trees owned by better-off households was 233, while poor households had an average of 148 trees, and the poorest households had only 40 trees. The results of the Kruskal-Wallis test indicate a significant difference in the ownership of trees under customary ownership by economic status (*p* < 0.001) (Fig. 3B). Pairwise comparisons using the Wilcoxon rank sum test show significant differences between the very poor and poor groups (*p* < 0.001), as well as between the very poor and better-off groups (*p* < 0.001), but not between the poor and better-off groups (*p* = 0.66).

**Table 6:** Distribution of trees under customary ownership on forested public lands with varying economic statuses

Economic Status	Households (N)	Number of trees under customary ownership (N)	Average number of trees under customary ownership (N)
Better-off	48	11,177	233
Poor	32	4725	148
Very poor	64	2554	40

The definitions of the categories of economic status are provided in Table S1

### Factors Influencing Participation in Forest Management Activities

Factor loadings were observed in all measured variables, indicating strong associations within the factor (Table 5). Specifically, attending meetings was associated with a load of 0.77, participation in emergency events showed a loading of 0.95, opinion sharing during conflict resolution had a loading of 0.99, and participation in forest protection activities and following recommended practices demonstrated a loading of 0.92. These high loads suggest that these activities are integral components of the latent dimension of community participation in PFM activities. The eigenvalue for the factor was 3.32, and the factor also explained 83% of the total variance.

The results of the OLS regression indicated that the number of trees in forested public lands under customary ownership, forestland used, caste, economic status, and displacement was statistically significant (*p* < 0.0001) and explained 30% (R^2^_adj._ 0.30) of the total variation in participation (Table 7). The number of forest trees (forstree) under customary ownership had a negative effect on participation in PFM activities, with additional trees associated with a statistically significant decrease in the participation rate of approximately 0.15 units (*p* = 0.01) when other variables in the model were controlled. This suggests that an increase in the number of trees under customary ownership correlates with reduced household participation in PFM activities. In contrast, the amount of forestland used (forsland) had a positive and statistically significant effect on participation. Households who used more forestland were more likely to participate in PFM activities, showing an increase of approximately 0.88 units (*p* = 0.02). Furthermore, households of Other Backward Classes tend to be significantly 0.74 units (*p* = 0.005) more involved in PFM activities compared to households of Scheduled Tribes.

**Table 7:** Relationship between explanatory variables and participation of households in participatory forest management activities

Variable	Coefficient (s.e.)
(Constant)	−0.58 (0.5)
Numbers of forest trees under customary ownership	−0.15 (0.06)*
Total household members	0.06 (0.05)
Average age of household	0.01 (0.01)
Age of household head	0 (0.01)
Number of school years attended in years by the household head	0 (0.02)
Agricultural land with legal title	0.15 (0.43)
Forest land used	0.87 (0.37)*
Total land with legal title	−0.09 (0.11)
Euclidean forest tree distance from owners’ residences	−0.0002 (0.0003)
Religion	
Hindu^r^	-
Christian	0.05 (0.17)
Caste	
Scheduled Tribes^r^	-
Particularly Vulnerable Tribal Group	0.19 (0.34)
Other Backward Classes	0.74 (0.26)**
Economic status	
Very poor^r^	-
Poor	0.56 (0.24)*
Better-off	0.67 (0.22)**
Primary livelihood	
Agriculture^r^	-
Forest-based activities	−0.31(0.28)
Migration	−0.06 (0.3)
Others	−0.18 (0.33)
Displacement	
No^r^	-
Yes	−0.83 (0.27)**
R^2^_adj._	0.30**

*, **, and *** indicate the statistical significance of the coefficients at 10%, 5%, and 1% level, respectively

Note: ^r^reference category

Economic status (econ) also played a crucial role in participation in PFM activities. Poor and better-off households showed a positive and significant influence on participation. Households classified as poor participated significantly 0.55 units (*p* = 0.02) more than very poor households when other variables were controlled. Similarly, better-off households engaged significantly 0.68 units (*p* = 0.002) more in PFM activities than very poor households. Furthermore, displacement status (displacement) had a negative and highly significant impact on participation, with displaced households 0.83 units (*p* = 0.003) less likely to participate in PFM activities than non-displaced households. These results highlight the importance of owning trees on forested public land under customary governance systems and various socioeconomic and demographic factors of their owners in shaping participation in PFM activities.

## Discussion

Our study quantitatively analyzes the roles of customary ownership over trees on forested public lands and various socioeconomic factors (e.g., caste, income, landholding, and displacements) in the context of the participation in PFM-related activities in Jharkhand, India. The study reveals disparities in participation rates. Households with more trees on forested public land under customary ownership participate less in PFM activities. Households belonging to other backward classes (higher caste) and better-off wealth groups participate more than Scheduled Tribe households and very poor households, respectively. Households that use more forestlands participate more in PFM activities. Finally, displaced households participate less in PFM activities than non-displaced households.

Our findings also reveal inequalities among wealth groups in study villages (Table S1). Better-off households have larger landholdings, have majority tree ownership on forested public land, and diversified income sources. These advantages enable better-off households to achieve greater food security and resilience. On the contrary, poor households face seasonal food insecurity due to smaller landholdings and less tree ownership on forested public lands and assets. Very poor households, primarily Mal Pahariyas, belonging to the Particularly Vulnerable Tribal Group, remain the most vulnerable. Due to displacement, they lack agricultural land and tree ownership on forested public land and are constrained by illness or absenteeism in their workforce; they depend heavily on welfare schemes, daily wage labor, and seasonal migration. These findings align with the existing literature on structural inequities in Jharkhand, a state characterized by the coexistence of abundant natural resources and widespread poverty (Sharma et al., 2024). Jharkhand’s Scheduled Tribe households suffer from persistent poverty, systemic corruption, and weak governance, which exacerbate inequalities (Sharma et al., 2024).

The relationship between economic status and participation in PFM is similarly complex, as evidenced by findings in existing studies and contexts. This study observed a positive association between higher economic status and participation, suggesting that wealthier households may have greater resources and flexibility to dedicate time and effort to PFM activities (Lise 2000). Others have observed negative or non-significant relationships (Oli and Treue 2015). This variability may be due to differences in the specific wealth indicators used (consumption, income, capital), regional contexts, and the nature of PFM programs. For instance, Dolisca et al. (2006) found that increasing annual income could enhance participation in participatory management processes, suggesting that wealth might be relevant in specific contexts where it facilitates engagement.

The main findings indicate that households holding customary ownership over a larger number of trees on forested public lands under customary ownership tend to participate less in PFM, compared to the study by Takahashi et al. (2024) in Ethiopia, which argued that a mixed private and community management system, including tree rights, can stimulate intensive forest management activities. This contrast is because households of study villages with more trees under customary ownership generate higher income through activities such as the cultivation of tasar silkworms in trees on forested public lands under customary ownership. Higher household income enhances affordability and the desire to invest in their children’s education and the family’s health care. The study villages lack basic education and a health care system. Therefore, the better-off households prefer to migrate to nearby cities such as Dumka, where those facilities are readily available. These findings are consistent with the findings of Sharma et al., 2024, who highlighted that tribal groups, including the Santhal and Mal Pahariya communities, bear the brunt of inequalities due to historical neglect and insufficient social infrastructure in Jharkhand. Similarly, previous studies have revealed that financially stable households prefer to migrate to nearer urban areas, offering quality education and health services owing to the lack of such services in villages (Bates and Carter, 1992; Varughese and Mukherjee, 2024).

More participation in the PFM by households with larger used forestlands is influenced by the household’s aspiration to legalize their ownership of used forestlands. This aligns with the FRA’s objective of recognizing and securing the rights of forest-dependent communities to traditionally used lands (FRA 2006). These findings are consistent with Ballal et al. (2023), who highlights that the FRA has transformed the status of forest dwellers from encroachers to legitimate owners with legal rights over forestland. This change empowers communities by granting them legal land ownership and improves their participation in forest management and conservation efforts through PFM.

Our findings indicate that a higher caste status is associated with increased participation in PFM activity. These findings are aligned with other studies conducted in other states of India, such as West Bengal, Bihar, Madhya Pradesh, and Nepal (Ghosh and Basu 2021; Lise 2000; Oli and Treue 2015). They have highlighted a positive and significant relationship between caste and the participation of forest users in PFM activities. However, the relationship between caste and participation is complex; for example, Lise (2000) and Bista et al. (2023) found that lower caste households participate more in forest management activities in Haryana, India, and the Middle Hills of Nepal. In Haryana, lower-caste households lack alternative sustainable livelihoods; therefore, they are highly dependent on forest products for their livelihoods due to limited access to alternative livelihoods. This dependency encourages their active participation in social fencing and water-sharing mechanisms facilitated by the state (Lise 2000). Bista et al. (2023) explained that participation of lower castes, including Dalits, has increased in PFM activities in Nepal’s Middle Hills, because the community forestry initiative offers training to build their skills, involving them in creating rules that address everyone’s needs and prioritizing their access to forest resources. However, in states like West Bengal and Bihar, higher caste groups dominate PFM due to greater access to resources and decision-making power. These findings underscore the need to analyze social hierarchies and institutional contexts to understand participation trends in PFM activities.

It is important to note that recognizing tree ownership in forested public land alone is insufficient in addressing systemic inequality where tribal communities face several technical, bureaucratic, transparency, weak governance, and human/capacity gaps. Such hurdles have made participation in the PFM more costly, especially for ultra-poor households. To overcome such challenges, nongovernmental organizations (NGOs) such as PRADAN have been involved in capacity-building and income-generating activities (Sharma et al. 2024). When the government is weak or stagnant in supporting such communities, other stakeholders who can bridge such gaps can be instrumental in increasing people’s participation in forest management activities and the economic growth of households. Barrow et al. (2016) highlight that forest tree rights are crucial for economic growth and integrating communities into the economic mainstream. They also emphasize that tree rights alone are insufficient; supportive regulations and capacity building are necessary to allow communities to effectively manage and benefit from these resources.

The study has certain limitations that must be acknowledged. We focused on only two villages, Dhawadangal and Sahritola, which can limit the generalizability of the findings to other regions with different sociocultural contexts. Furthermore, our study did not extensively consider historical factors, long-term changes in forest management practices, and community dynamics that could influence current participation and socioeconomic conditions. Despite these limitations, this study contributes valuable dimensions to the existing literature. Future research could explore several possibilities to build on these findings. Expanding the geographical scope to include many villages in different regions of Jharkhand or other states could provide a more comprehensive understanding of the influence of customary ownership over trees of forested public lands on the participation of PFM.

## Conclusion

Our study offers unique insights into the relationship between private ownership over trees located on forested public lands as common property and collective management of privately owned common property. To our knowledge, this is the first study that uses a quantitative approach to explore the relationship between customary ownership over trees on forested public land and participation in PFM activities. Socioeconomic factors such as caste, economic status, and displacement status of households result in different levels of participation in PFM activities. The findings highlight the importance of addressing social and caste inequalities and providing the necessary attention and support to displaced communities, such as Mal Pahariyas, to improve the participation of the most vulnerable and marginalized communities in PFM activities. The study also underscores the critical role of tree-based livelihood improvement and customary governance systems in advancing sustainable forest management.

The 2023 NWPC recognizes the importance of integrating customary practices and traditional knowledge. Similarly, the 2014 NAP focuses on integrating trees with agricultural practices to improve productivity and support the livelihoods of forest dwellers. Our key findings on customary ownership of trees on forested public lands highlight the factors, such as the number of trees under customary ownership, socioeconomic status, and wealth, that can be incorporated into such policy instruments. Recognizing and incorporating customary ownership over trees on forested public lands can assist in addressing several governance challenges and apprehensions related to the amendment of the 2023 FCA. Granting customary ownership of trees to local communities while keeping forest land under the ownership of the Government of India reinforces the decision-making power of local institutions and strengthens decentralized governance. Our findings on customary tree ownership on forested public lands are in agreement with the Supreme Court’s 1996 T.N. Godavarman Thirumulpad v. Union of India ruling, which emphasizes the integral relationship between forest land and its tree cover (Godavarman 1996). By giving communities, a stake in the management of trees under customary ownership on forested public land, this framework mitigates power imbalances, secures local livelihoods, and improves forest health and community well-being while promoting and supporting PFM in India and, hopefully, beyond.

## Supplementary information

Supplementary materials

## Data Availability

The data that has been used is confidential.
