# Supplementary material for: Assessing the Impact of Customary Ownership of Trees and Socioeconomic Factors on the Participatory Forest Management in Jharkhand, India
Source: Environ Manage. 2025 Feb 22;75(5):1201–15. doi: 10.1007/s00267-025-02129-x (PMC12033090; doi:10.1007/s00267-025-02129-x)
Supplement: Supplementary file 1 — Supplementary materials [file 267_2025_2129_MOESM1_ESM.docx]

**Title**

Assessing the impact of customary ownership of trees and socioeconomic factors on the participatory forest management in Jharkhand, India

**Supplementary materials**

Table S1. Characteristics of the wealth group.

| Wealth Group 1  (Rich) | Wealth Group 3  (Poor) | Wealth Group 4  (Very Poor) |
| --- | --- | --- |
| - Food security for more than six months - Landholding between 0.20 ha and 0.60 ha. - Own a good number (5 - 10) of livestock, including cows, goats, and hens. - One or more members of the household work in government or private organizations or have a business. - The main livelihoods include agriculture, silkworm cultivation, and small business. - Very few members migrate to urban cities to earn a living. - One of the household members: village head, ward member, retired government officer, Anganwadi member, etc. - Have some household assets such as motorcycles and televisions. - Annual household income between Rs.60,000 and Rs.150,000. | - Food security for five to six months with seasonal food insecurity - Landholding between 0.20 and 0.08 ha. - Own a small number (less than 5) of livestock, including goats and chicks. - Very few assets. - The main livelihoods are migration, tasar silkworm cultivation, agriculture in small plots, fishing, and wage labour. - Annual household income between Rs.40,000 and Rs.60,000. | - Food security for less than four months and is heavily on government welfare schemes. - There was no land for agriculture, but they received land from the government to construct their houses. - Lack of livestock. - Primary sources of income include migration, fishing, and wage labor. - Limited assets and shortage of necessities. - The adult workforce is weakened by death, absenteeism, or chronic illness. - The household workforce mainly comprises children, women, and the elderly, who command a low daily wage. - Annual household income less than Rs.40,000. |
